# Data-Driven Modeling to Assess Receptivity for Rift Valley Fever Virus

**DOI:** 10.1371/journal.pntd.0002515

**Published:** 2013-11-14

**Authors:** Christopher M. Barker, Tianchan Niu, William K. Reisen, David M. Hartley

**Affiliations:** 1 Center for Vectorborne Diseases and Department of Pathology, Microbiology, and Immunology, School of Veterinary Medicine, University of California, Davis, Davis, California, United States of America; 2 Fogarty International Center, National Institutes of Health, Bethesda, Maryland, United States of America; 3 Division of Integrated Biodefense, Georgetown University Medical Center, Washington, District of Columbia, United States of America; 4 Department of Microbiology and Immunology, Georgetown University Medical Center, Washington, District of Columbia, United States of America; United States Army Medical Research Institute of Infectious Diseases, United States of America

## Abstract

Rift Valley Fever virus (RVFV) is an enzootic virus that causes extensive morbidity and mortality in domestic ruminants in Africa, and it has shown the potential to invade other areas such as the Arabian Peninsula. Here, we develop methods for linking mathematical models to real-world data that could be used for continent-scale risk assessment given adequate data on local host and vector populations. We have applied the methods to a well-studied agricultural region of California with 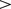
1 million dairy cattle, abundant and competent mosquito vectors, and a permissive climate that has enabled consistent transmission of West Nile virus and historically other arboviruses. Our results suggest that RVFV outbreaks could occur from February–November, but would progress slowly during winter–early spring or early fall and be limited spatially to areas with early increases in vector abundance. Risk was greatest in summer, when the areas at risk broadened to include most of the dairy farms in the study region, indicating the potential for considerable economic losses if an introduction were to occur. To assess the threat that RVFV poses to North America, including what-if scenarios for introduction and control strategies, models such as this one should be an integral part of the process; however, modeling must be paralleled by efforts to address the numerous remaining gaps in data and knowledge for this system.

## Author Summary

Rift Valley fever virus is a pathogen enzootic to sub-Saharan Africa, with epidemic transmission occurring sporadically between mosquitoes and mammals, notably livestock. The virus is regarded as a global threat to agriculture and human health because it has proven capable of expanding its range into western and northern Africa, Madagascar, and the Arabian Peninsula, and a recent study has shown that mosquitoes in North America are capable of transmitting the virus. Here, we used a set of mathematical equations to formulate a logical representation of potential transmission mechanisms, and we informed the model with real-world data and generalizable methods to define spatial and temporal variation in mosquito and host abundance. We applied these methods in California's warm, agricultural Central Valley, an area with a history of mosquito-borne virus transmission and a hub of California's dairy industry. Model-derived transmission estimates indicated broad potential for transient epidemics that could result in economic losses in livestock in all but the coldest winter months, but the greatest risk for intense, sustained transmission occurred during the summer when both vector abundance and temperatures were highest. We also highlight critical gaps in the data available to inform models for Rift Valley fever virus.

## Introduction

Rift Valley fever virus (RVFV; viral family *Bunyaviridae*, genus *Phlebovirus*) is a pathogen that causes febrile illness in domestic ruminants (sheep, cattle, and goats) and humans throughout Africa and parts of the Arabian Peninsula [1]–[3] that may be transmitted by several genera of mosquitoes [3]–[6]. Outbreaks often result in heavy economic costs through loss of livestock, especially when associated with an incursion into a new area [7], [8]. Although never detected in the Western Hemisphere, RVFV is a threat to human and livestock health in North America and is included on select agent lists of the U.S. Department of Health and Human Services and the U.S. Department of Agriculture [9].

Mosquito species found to be vectors of RVFV with varying degrees of efficiency in laboratory settings [6], [10] are known to be present throughout much of the U.S.[11], but other aspects of potential transmission cycles remain inadequately studied. To properly assess and mitigate the risk posed by a RVFV invasion, methods are needed to identify areas that are most likely to support transmission, the time periods when transmission is expected to pose a risk, and whether an introduced virus could become established. To date, such questions have been addressed by only a few analytic methods, including expert elicitation [12], basic GIS overlays of humans and vectors with a hypothetical host [13], and pathways analysis [14]–[16].

Process-based mathematical models provide a useful platform to coalesce disparate data, make logical assumptions concerning data gaps, and evaluate a range of potential scenarios. Gaff et al. developed a dynamical model for RVFV [17] that included livestock hosts and two genera of mosquitoes, *Aedes* and *Culex*, that respectively were or were not capable vertically transmitting RVFV. This model's structure has been extended in several important ways to 1) accommodate spatial structure through host or vector movements [18], [19], 2) assess potential control methods [20], [21], or 3) include humans [22] or asymptomatic livestock hosts [23]. These models have resulted in important advances in modeling RVFV, but their application is limited by the lack of appropriate data to inform parameters, many of which have been recycled between models, defined arbitrarily, or borrowed from literature on other arboviruses that may not apply for RVFV.

In the current study, we apply a unique and generalizable approach that links real-world data with the mathematical models, utilizing broadly available national-scale data where possible. To illustrate the methodology, we consider results for the southern Central Valley in California, an area with large, well-documented host and vector populations. We present two model-derived transmission metrics that quantify expectations for typical (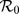, [24]) and maximal (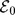, [25]) transmission from an initial disease-free state, and we map these metrics according to the spatial pattern of vector abundance associated with various land uses. We also show how these metrics change in time as a function of temperature, thereby enabling the assessment of seasonal transmission risk. We also highlight several critical data gaps that must be addressed.

## Methods

### Study area

**Figure 1:**
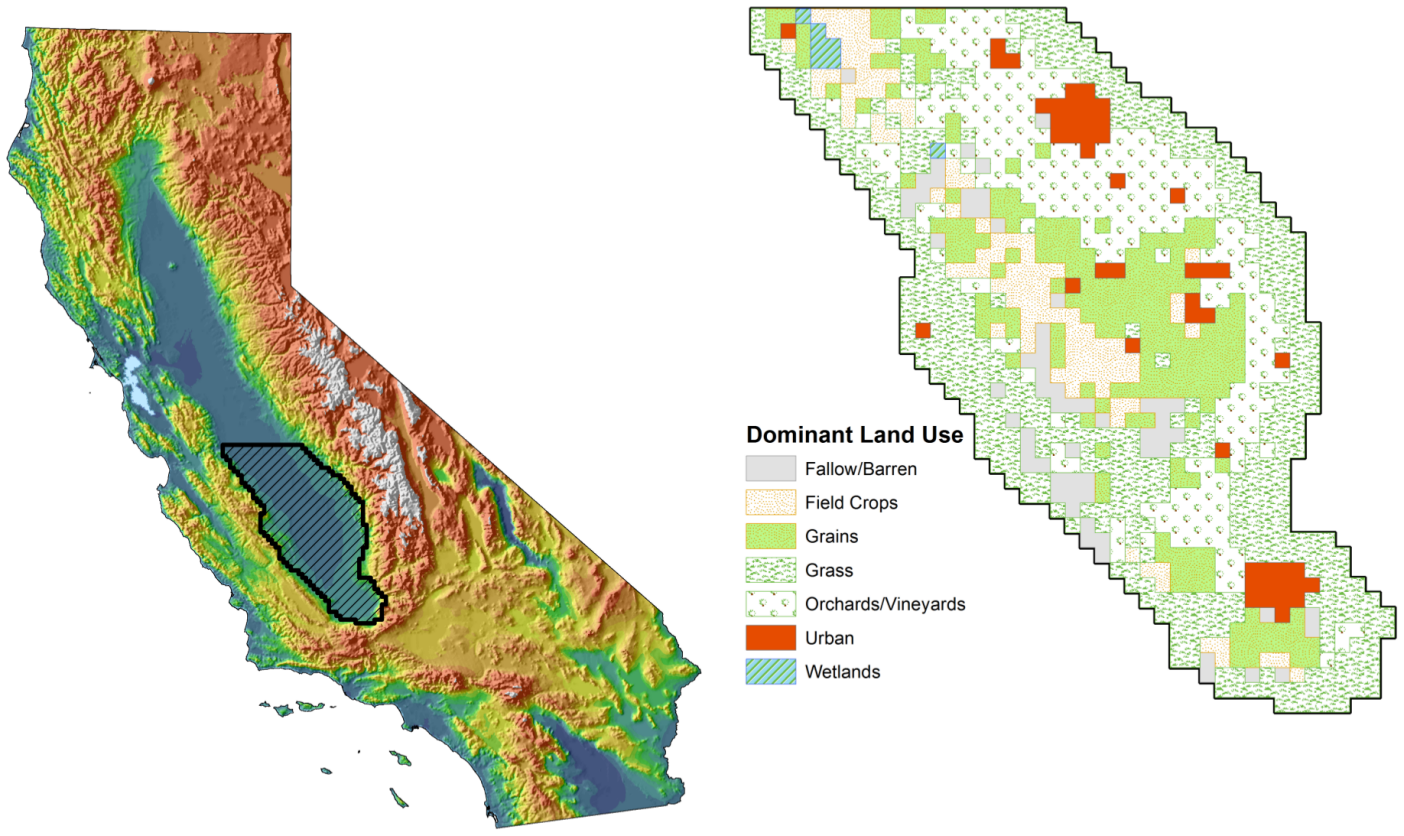
Study area. Map showing the location of the study area within California (left panel), and a map of the study area depicting the dominant land use within each 5-km grid cell (right panel).

California's Great Central Valley extends for 700 km north to south through the center of the state and is home to extensive and varied agricultural lands that include irrigated crops, livestock operations, natural or restored wetlands, and urbanized areas. In this study, we considered the southern half of the Central Valley (Figure 1), which contains very high densities of livestock (primarily dairy cows, with 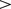
1 million cattle within the study area) interspersed with managed wetlands and multicrop agriculture that can produce large populations of competent vectors (e.g., *Culex tarsalis*). Deer were rare on the valley floor and generally were restricted to surrounding higher-elevation foothills and mountains, and sheep typically were moved into the valley for grazing only during the cooler months of the year when transmission was expected to be minimal. The area is likely to be climatologically permissive for RVFV transmission as this is the warmest part of the valley and supports consistently high transmission of West Nile virus [26] and, previously, other arboviruses [27]. For the model, appropriate spatial dimensions were needed for patches that would represent the heterogeneity in land cover and host and vector densities at a fine enough scale that populations could be assumed to be well-mixed, given described ranges of vector movement [28]. To achieve this, we defined a uniform grid of 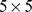 km squares (25 km^2^) that covered the study area, and all model input variables were scaled to this grid. All model outputs were calculated by grid cell for each day of the year.

### Data

#### Temperature

**Figure 2:**
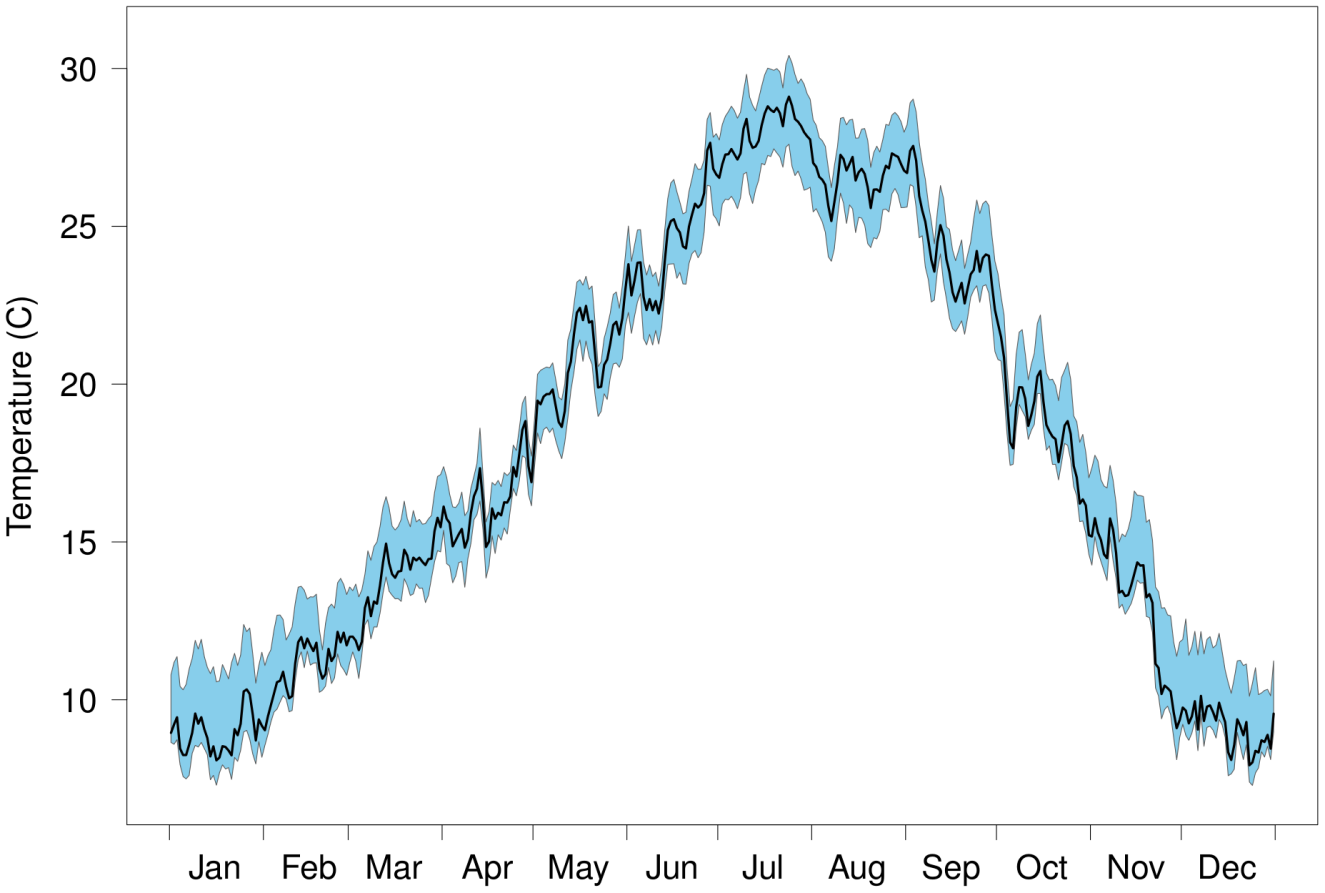
Seasonal temperature pattern within the study area. Graph showing daily mean temperatures (dark line) and 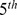
–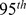 percentiles (shaded area) for the study area.

Daily maximum and minimum temperatures were acquired from the National Aeronautics and Space Administrations Terrestrial Observation and Prediction System (TOPS; http:// ecocast.arc.nasa.gov) [29], which uses weather and ecosystem models to combine ground-based and remotely sensed inputs to generate multiple measures of environmental conditions. For this study, the TOPS surfaces (1 km^2^ resolution) for daily mean temperatures were averaged for each day of the year for the most recent 10-year period (2002–2011) and spatially within each 25 km^2^ grid cell so that temperatures represented the typical pattern for each spatial location, summarized in Figure 2.

#### Vectors

Mosquitoes in the genera *Aedes* and *Culex* are important vectors of RVFV in enzootic areas, and we focused on two species that are both abundant within our study area [26], [30] and likely to be capable of transmitting RVFV, albeit to differing degrees [6]. *Aedes* mosquitoes can be infected with RVFV either vertically from infected females [31] or horizontally via a blood meal from an infectious host. We focused on *Aedes melanimon* because it has been an important vector of vertically maintained California encephalitis virus [32], frequently feeds on mammals [33], and is likely to be a low-competent horizontal vector of RVFV based on results for the closely related species, *Aedes dorsalis* [6]. *Culex* vectors are able to transmit RVFV horizontally, but not vertically to their offspring, and here we considered *Cx. tarsalis*, which is a principal vector of several encephalitis viruses [34], [35], feeds opportunistically on both birds and mammals [36], [37], and is the most competent laboratory vector of RVFV studied in North America [6].

**Figure 3:**
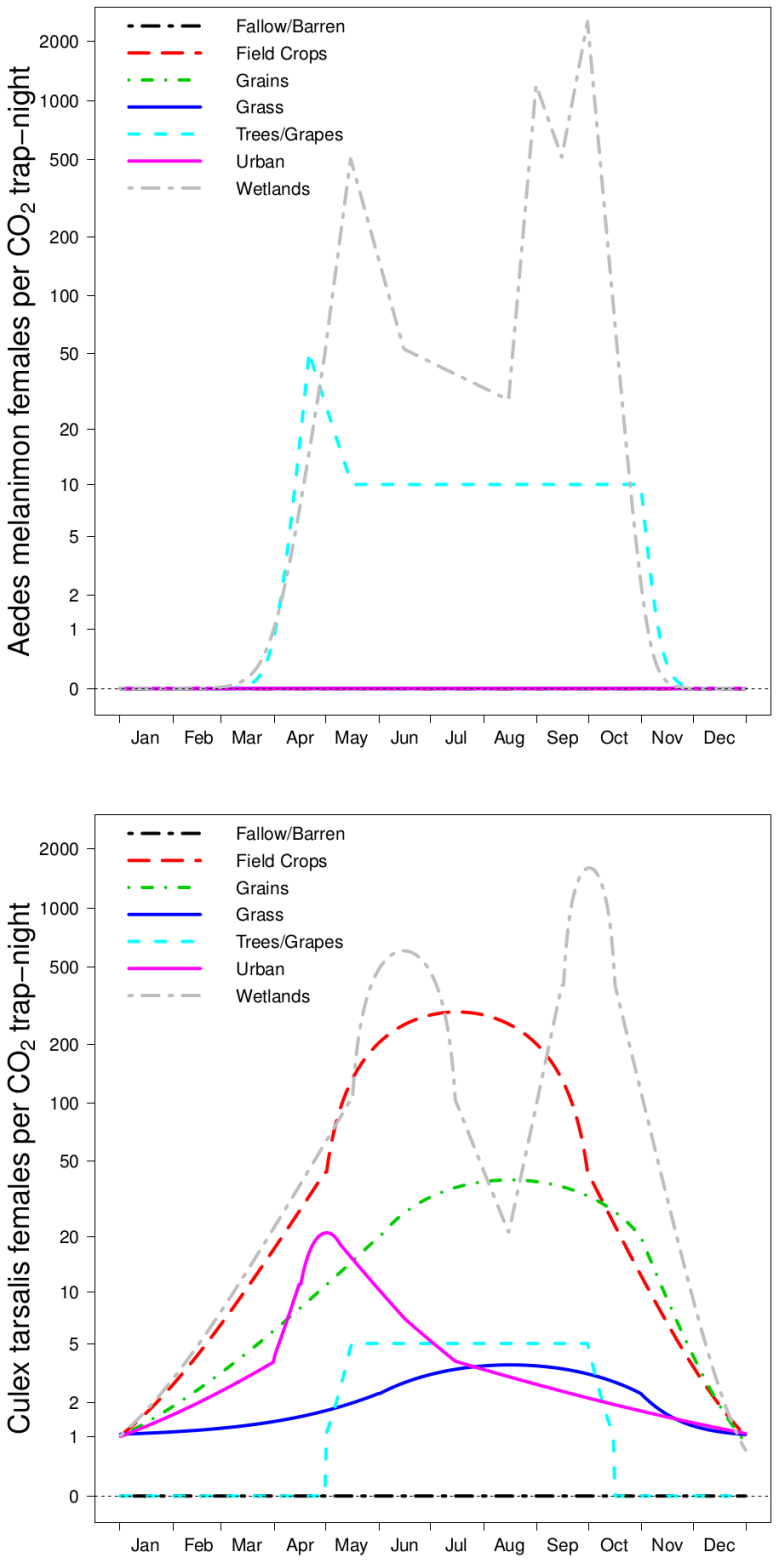
Seasonal mosquito abundance patterns. Realistic annual patterns for *Cx. tarsalis* and *Ae. melanimon* defined using trap data for each of the dominant land use categories within the study area. Traps collected *Ae. melanimon* only in 2 land uses, with the largest numbers occurring in seasonally flooded wetlands.

Annual patterns for the relative abundance of these vectors were assigned to each of several broad land use classes representing a generalization of the narrowly defined single-crop classes in the most recent USDA Cropland Data Layer for 2011 (Figure 1; http://www.nass.usda.gov/research/Cropland/ SARS1a.htm). Our goal was to represent typical variation in daily abundance for each of the two mosquito genera in each land use class based on data from dry ice-baited CDC-style traps [38] operated by vector control agencies within the study area. Averaging trap counts spatially or over several years would not achieve this goal, especially for *Aedes* populations that hatch synchronously in response to flooding of eggs, resulting in an abundance spike when the cohort emerges. Such sharp peaks occur at similar but slightly different dates each year, with the result that a multi-year average would smooth the annual spikes into a rounded peak that is not representative of any year. To avoid this problem, a small number (3 to 5) of representative CO_2_-baited trap sites were identified within each land cover class, and their time series of trap counts were used to define a “consensus” time series for each class that captured the key features of its annual pattern (namely, seasonally varying rates of increase and decrease, and abundance minima and maxima; Figure 3). To apply these patterns spatially, each spatial grid cell was characterized by its dominant (i.e., most common) land cover class, and the relevant abundance patterns for *Aedes* and *Culex* were applied, multiplied by the gonotrophic period to scale the fraction of vectors that would be host-seeking on a given night to the total population size and by each grid cell's respective total host abundance (cows+birds) as defined below, based on the assumption that a trap represents one host (also see Mathematical Appendix Table S2 in Text S1). The scale factor for the gonotrophic period was used because traps represent only the fraction of all females that seeks hosts on a given night, equal to 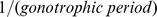 on average, but the model requires an estimate of the total female population, including those in other stages of the gonotrophic cycle (e.g., resting or egg-laying).

#### Livestock

RVFV antibodies and some viral isolates have been observed in a variety of African mammals, but viremias sufficient to infect biting vectors have been reported only in cattle, sheep, and goats [1], although high viremias may also occur in humans [39]. Our model includes two host populations, with the first represented by dairy cows considered to be competent for transmitting RVFV, and the second represented by birds considered to be incompetent “sink” hosts. Within our study area, dairy cows were the most abundant ungulates, and we calculated the total number of cows within each grid cell using data on dairy sizes obtained from the California Environmental Protection Agency's Central Valley Regional Water Quality Control Board.

#### Birds

*Cx. tarsalis* also feeds opportunistically upon birds [36], [37], and we expected birds to be likely alternate hosts to represent the incompetent class. We acknowledge that *Ae. melanimon* also feeds frequently on hares and rabbits (Order Lagomorpha [33]), but data on their abundance within our study area were not available. To quantify the abundance of birds, we considered seven species of birds that are abundant near dairies and could divert bites from cattle: Red-Winged Blackbirds (*Agelaius phoeniceus*), Brown-Headed Cowbirds (*Molothrus ater*), Brewer's Blackbirds (*Euphagus cyanocephalus*), House Finches (*Haemorhous mexicanus*), House Sparrows (*Passer domesticus*), European Starlings (*Sturnus vulgaris*), and Rock Pigeons (*Columba livia*). Gridded data from the USGS Breeding Bird Survey (BBS; 21.5 km resolution) were downscaled to our 5-km grid by calculating a weighted average of the per-BBS route bird abundance for the species above within a 10-km buffer around each 5-km grid cell. Next, we rescaled per-route bird abundance to an estimate of the total birds within each grid cell by multiplying the per-route values by the ratio (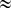
4) of estimated areal coverage of BBS routes to the area of the grid cell. BBS routes consist of 50 stops, spaced 800 m apart, with observers attempting to detect all birds within a 0.4-km radius at each stop; assuming they effectively observed half that radius (see discussion of detection in [40]), the area observed would be 6.28 km^2^.

### Mathematical modeling

**Figure 4:**
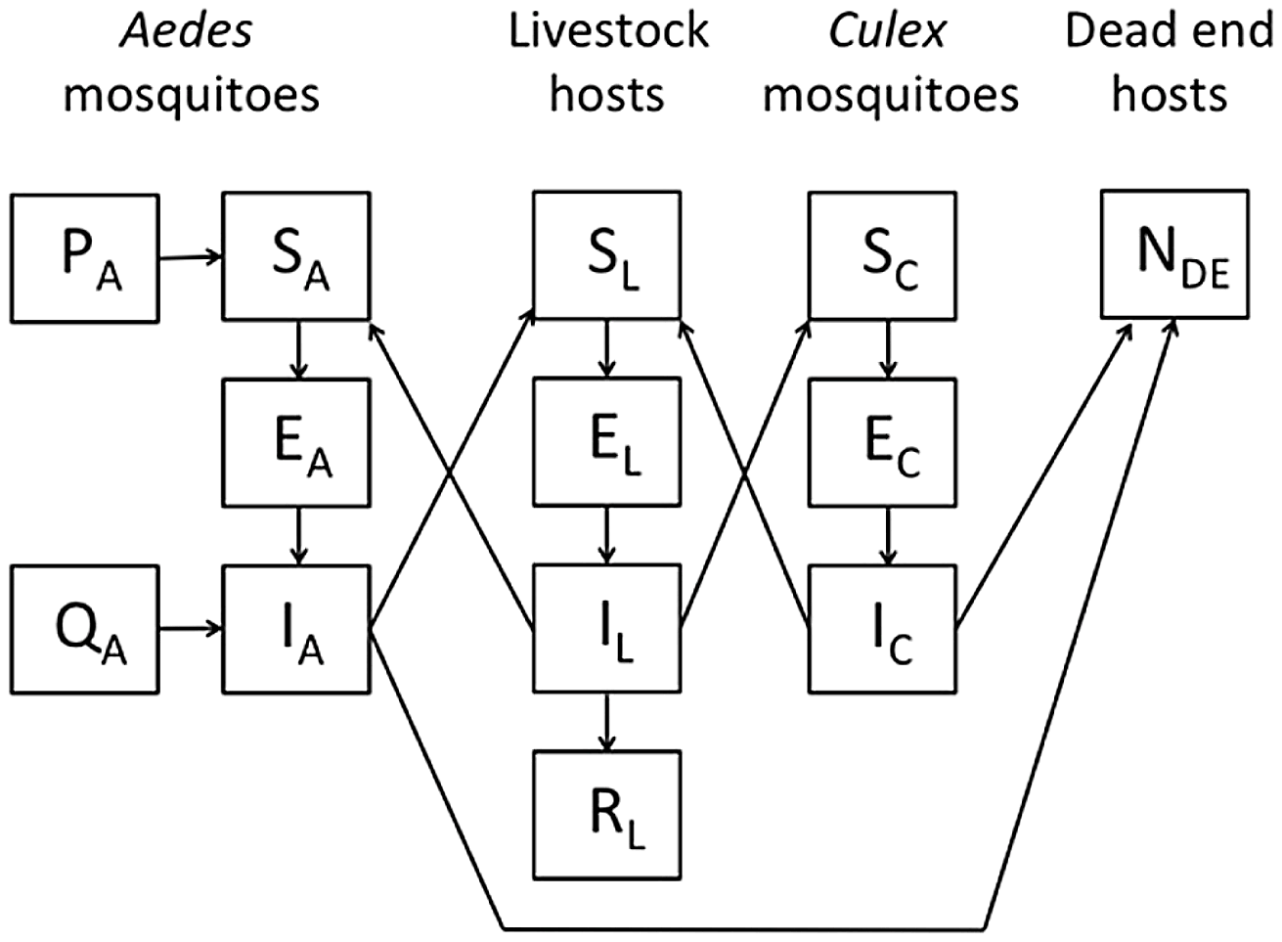
Diagram of the model. Schematic of the SEIR model constructed for Rift Valley fever virus circulation in California. Mosquitoes are categorized as capable of vertical transmission (*Aedes*) or not (*Culex*). For *Aedes*, adult mosquitoes emerge from uninfected (P) or vertically infected (Q) eggs. Hosts are categorized as highly competent (livestock) or incompetent (dead-end hosts) for RVFV transmission. See the text for a complete explanation.

Process-based, dynamical mathematical models of virus transmission are built from knowledge of the interactions among virus, hosts, and vectors. In the case of RVFV in North America, such issues are uncertain. In the current study we extend previous work [17], [41] to construct a mathematical model of RVFV (Figure 4), with the following assumptions regarding the anticipated epidemiology of RVFV in California.

1. *Hosts.* We consider one prototypical competent host, which corresponds to domestic ungulates. Incubation periods and peak viremia in cattle, goat, and sheep species are similar, so the generic host could be any of these animals. Competent hosts become infected when fed upon by infectious vectors. Such hosts may then die from RVFV infection or recover, whereupon they have lifelong immunity. Incompetent or dead end hosts may be either mammals or birds, and are fed upon by vectors but do not become infectious and therefore serve as a sink for the virus and dampen transmission.
2. *Vectors.* We included two types of vectors, with the first (typified by *Ae. melanimon*) capable of vertical transmission to offspring and low-level competence for horizontal transmission, and the second (typified by *Cx. tarsalis*) being a highly competent horizontal vector that does not transmit the virus vertically. Vertical transmission of RVFV has been observed in field-collected larvae [31], but laboratory data from related viruses show a complex picture of the efficiency of vertical transmission. The literature suggests that vertical transmission is a low-frequency event in nature on average. Therefore, we included vertical transmission in the model at low rates of occurrence.
3. *Vector host selection*. Vectors bite either RVFV competent or incompetent hosts in proportion to total host population size; i.e., we assume that there is no mosquito feeding preference. Particularly for *Ae. melanimon* that feeds primarily on mammals, this assumption may seem questionable, but the very high densities of cattle in our study area ensured that they were the predominant bloodmeal source even with opportunistic feeding (cattle represented the majority of hosts in 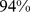 of grid cells and 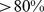 of hosts in 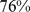 of grid cells).
4. *Host states.* At any given time, competent hosts are either susceptible (S) to infection, infected but not infectious (i.e., they possess a latent infection, E), infectious with RVFV (I), or immune following recovery from infection (R). If a competent host survives, after clearing infection, it is assumed to retain immunity for life.
5. *Vertical transmission. Aedes* mosquito eggs are either uninfected (P) or infected (Q) when laid and mature into either susceptible (S) or infectious (I) adults, respectively.
6. *Horizontal transmission.* At any given time, adult mosquitoes are either susceptible (S) to RVFV infection, infected but not infectious (E, during the extrinsic incubation period), or infectious with RVFV (I). Once infectious, mosquitoes are assumed to remain infectious for life.
7. *Population dynamics.* The growth of all vector and host populations is logistic and characterized by their respective rates of birth and non-disease related mortality. Livestock hosts can die from disease resulting from infection with RVFV. Mosquito population dynamics are defined by trap data in relation to land use as described above.

Temperature dependence of the vectors' extrinsic incubation rate (i.e., the inverse of the extrinsic incubation period, EIP) and the gonotrophic cycle length (gonotrophic period, GP) were modeled based on published data as follows. We digitally extracted data points for temperatures at which a median EIP could be estimated from Figure 3 of [42] (26 and 33

C) and Figure 1 of [43] (17 and 28

C). Logistic curves were fitted to the proportion of mosquitoes with disseminated infections over time for experiments with *Ae. fowleri* and *Ae. taeniorhynchus*. To our knowledge, these studies are the only published experiments that explore the temperature-vector competence relationship for RVFV. From the resulting model functions, we estimated the EIP as the median time to disseminated infection of RVFV in the mosquito. Carrying out a linear regression on the rate as a function of temperature resulted in the model: EIP =  (0.007084 × temperature−0.103820)^−1^. The relationship between EIP and temperature also has been studied for *Culex* mosquitoes [42], [44], but heterogeneity among experiments and a paucity of comparable data points precluded construction of an analogous EIP model for this genus. Therefore, we modeled extrinsic incubation in *Culex* and *Aedes* by the same function. The gonotrophic period (i.e., the number of days between bloodmeals) was modeled as *GP* =  2+(−0.066+0.018 × temperature)^−1^, using a published linear regression equation for the ovarian maturation rate [45] plus 2 days for oviposition and locating a bloodmeal host. The environmental carrying capacity could not be explicitly measured, and for both vectors, it was approximated daily using the vector abundance for the following day based on the typical abundance time series described above. This resulted in the desired inflation and deflation of the density-dependent birth rates in proportion to the rate of population growth or shrinkage, respectively, with a corresponding inverse impact on death rates.

We implemented the full model using a set of ordinary differential equations (mathematical details appear in the appendix). Using the methods described in van den Driessche and Watmough [46], we derived an expression for the basic reproduction ratio, 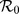, which represents the average number of secondary infections that arise from a single infectious individual (vector or host) introduced into a completely susceptible population[24], [47], so that when 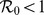, there are insufficient new cases per case for propagation and the pathogen cannot persist in the population. When 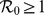, the pathogen is efficiently transmitted and becomes enzootic; elevated 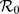 values indicate that transmission is more intense and that stochastic fadeout of the pathogen is less likely. For complex models of vectorborne infections, it has been demonstrated that outbreaks are possible for 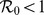 under certain circumstances [48], [49]. Because the model incorporates both vertical and horizontal transmission, 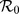 was written as the sum of the 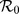 values for each mode of transmission determined separately, 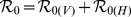 [17], [18], [50]. Details of the 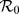 computation and a sensitivity analysis of the model appear in the Mathematical Appendix.

In addition to 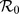, we computed a recently described, metric, 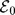, that quantifies the reactivity, or epidemicity, of the system [25]. 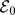 represents the maximum number of new infections produced by an infective individual at a disease free equilibrium, and like 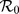, epidemicity is a threshold quantity; when 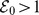, transient epidemics (i.e., outbreaks that may eventually fade out) are possible regardless of the average system behavior predicted by 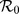. When 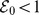, transmission, even for brief time periods, is not expected. Evaluating 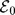 from our model allows us to investigate the potential for RVFV outbreaks in areas and times when 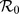 suggests that efficient transmission is not possible. 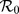 and 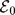 are both functions of the model parameters shown in Mathematical Appendix Table S1 in Text S1.

Stochastic sampling from biologically relevant ranges of parameters was used to assess the sensitivity of 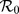 and 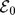 to the model parameters. The ranges for each parameter are presented in Mathematical Appendix Table S3 in Text S1. 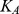 and 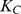, the vector carrying capacities, were computed from observed data as described above. Likewise, the EIP and vector GP values were functions of temperature. We assumed a uniform distribution for each parameter across ranges shown in Mathematical Appendix Table S3 in Text S1. The ranges of all the other parameters are from the references shown in Mathematical Appendix Table S1 in Text S1. Our model includes 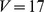 uncertain variables, so 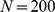 sets of sampled parameter values were generated by Latin hypercube sampling following the suggestion of Matala [51] that an 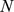 such that 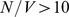 should suffice for the number of stochastic samples of complete parameter sets. Partial rank correlation coefficients (PRCC) were computed across ranges of parameters described in Mathematical Appendix Table S3 in Text S1 to assess the significance of each parameter with respect to 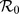 and 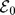.

Spatial analysis of temperatures, land cover, and host and vector abundance was carried out using R version 2.15 [52], ArcGIS 10.0 (ESRI, Redlands, CA, USA), and PostgreSQL 9.0 (http://www.postgresql.org) databases with added spatial capabilities of PostGIS (http://postgis.refractions.net). All code for mathematical modeling was written in 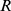 version 2.15 [52].

## Results

### Potential for establishment

**Figure 5:**
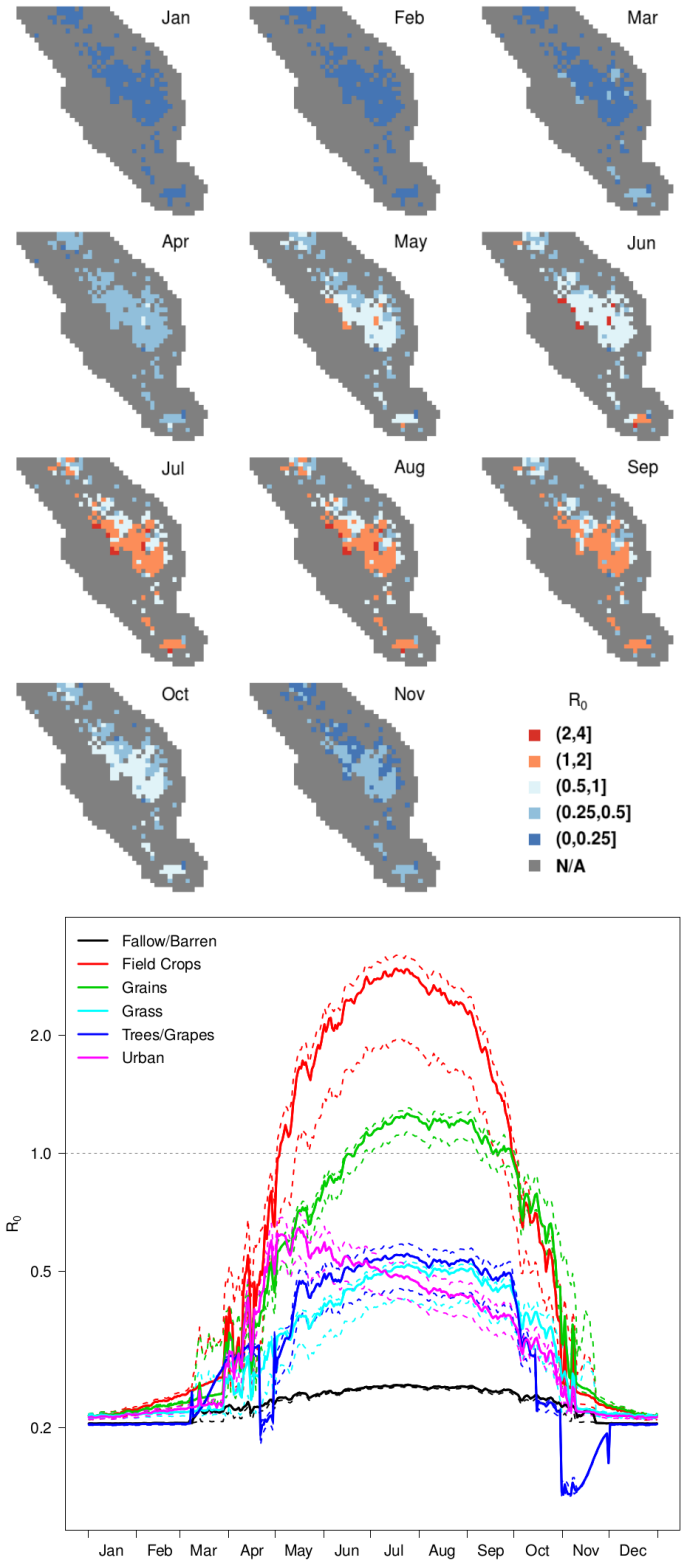
Spatio-temporal patterns in the basic reproductive ratio, 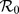. Maps (upper panel) showing the mean basic reproductive ratio, 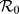, by month, and a graph of median daily 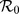 values (lower panel) by land use class. Dashed lines in the lower panel indicate the 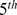 and 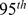 percentiles for the land use class of the same color. Wetlands and other grid cells without competent hosts (i.e., dairy cows) are mapped in gray and were not included in the analysis because transmission would not be expected in those locations. December is omitted from the maps because it did not differ meaningfully from January, with 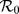 universally 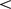
1.

The seasonal patterns for the basic reproductive ratio for RVFV (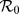; Figure 5) indicated that the risk for sustained transmission increased rapidly by May, with 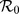 exceeding 1 in the areas with both cattle and field crops (Figure 5). Initially, these areas at risk consisted of a small number of grid cells in the center of the study area and a single cell near the southernmost end of the valley that could serve as early foci for transmission, and these areas remained at higher risk than other areas through the summer. Risk was greatest overall from late June–September, when a much broader area was at risk for sustained transmission that included grains and field crops and covered Tulare County, the core of California's dairy industry. In all areas, 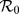 values 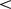
1 from October–April indicate that introductions from late fall–early spring would be unlikely to become established and that persistence of RVFV through winter may depend on mechanisms for long-term maintenance between epidemics (e.g., vertical transmission in vectors).

### Potential for outbreaks

**Figure 6:**
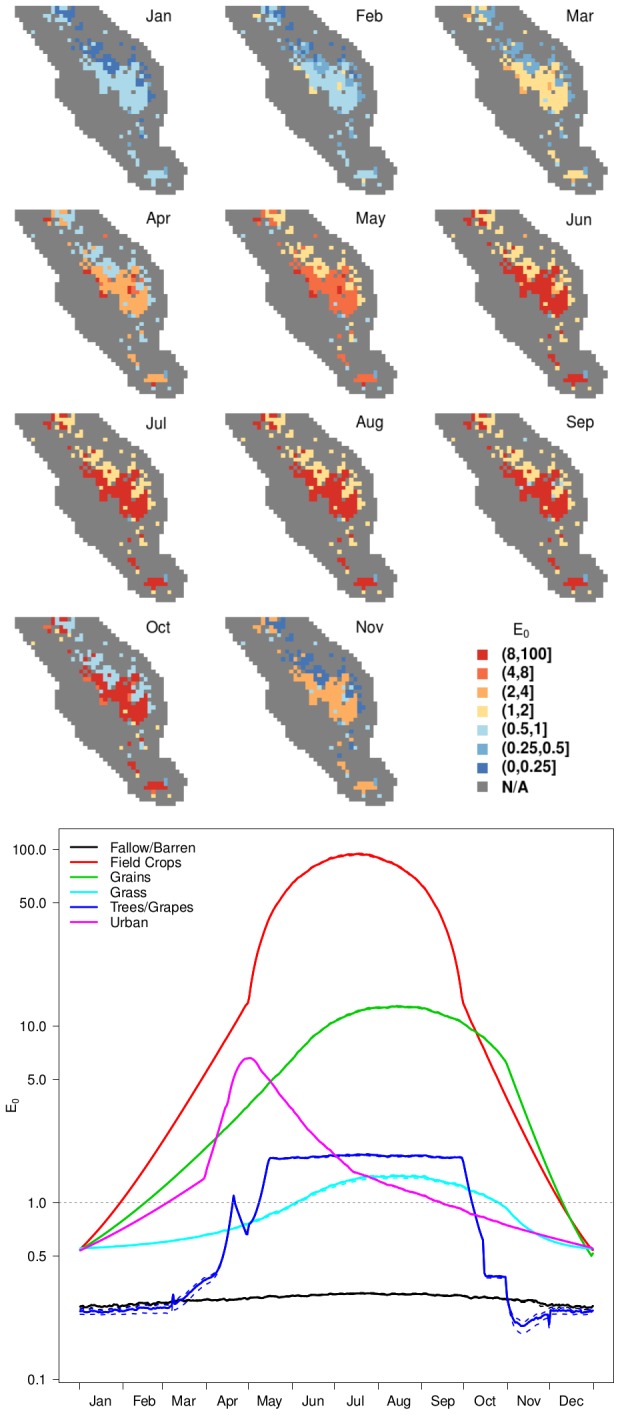
Spatio-temporal patterns of epidemicity, 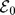. Maps (upper panel) showing an estimate of the maximal transmission potential, 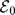, by month, and a graph of median daily 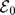 values (lower panel) by land use class. Dashed lines in the lower panel indicate the 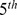 and 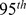 percentiles for the land use class of the same color. Wetlands and other grid cells without competent hosts (i.e., dairy cows) are mapped in gray and were not included in the analysis because transmission would not be expected in those locations. December is omitted from the maps because it did not differ meaningfully from January, with 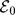 universally 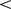
1.

Our estimates of epidemicity for RVFV, 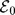, were much higher than the average expectations of 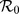 (max = 95.3 and 3.2, respectively) and indicated that transient outbreaks could occur over a broader spatio-temporal window than that circumscribed by 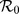 alone (Figure 6), although the relative seasonal patterns for the two metrics were strongly correlated (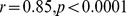). 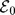 values 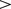
1 indicate that transmission was possible from February–November in agricultural areas, although the number of cases probably would remain small for introductions during February–April or November. The highest transmission potential (Figure 6) occurred in places and times where cattle were present and vectors reached high densities. Specifically, 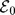 values were greatest in areas dominated by field crops and grains, which generally had the highest combined concentrations of *Culex* vectors and cattle in the study area, although the latter had little impact on epidemicity. Risk of RVFV transmission in urban areas was somewhat lower, with the highest level expected during spring, associated with the typical peak in *Culex* abundance at that time, followed by a slow decline in both 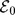 and vector abundance through the end of the summer. Orchards and vineyards (trees/grapes) and grasslands had low risk for epidemics due to their low vector abundance, and fallow or barren habitats had no risk for outbreaks, even when cattle were present. In all areas, 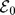 was closely linked to the abundance and carrying capacity of vectors, and 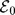 was greatest during spring and summer when the abundance of vectors, primarily *Culex*, was highest. For the scenarios under study, other parameters had little impact on 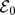, resulting in negligible variation around the average seasonal pattern for each land use class (Figure 6).

### Wetlands

Seasonally-flooded wetlands were not included in our analysis because the relatively small fraction of the total study area that they occupied (Figure 1) did not include dairy cattle. However, these areas deserve special consideration because they were the only areas where both *Aedes* and *Culex* reached high abundance (Figure 3), making them a reasonable analogue to the dambo habitats where RVFV is enzootic in east Africa, although the timing of their flooding is linked to human water management rather than rainfall. If dairy cattle or other competent hosts were present, both horizontal and vertical transmission would be possible, presenting strong potential for both transient epidemics and establishment. To evaluate this possibility, we calculated 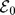 and 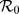 for the wetlands in the study area (Figure 1), with the addition of 1,000 cattle to each grid cell to simulate the effect of a moderate-sized dairy adjacent to the wetlands (median dairy size for the study area
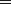
1,140 cattle). Transmission potential was higher than for other land uses and reflected the abundance of *Culex* vectors as before. There were two annual peaks of 186 on June 14 and 489 on September 30 for 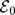 and 2.84 on June 27 and 4.37 on September 27 for 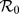.

### Sensitivity analysis

Sensitivity analysis revealed that both 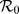 and 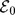 were particularly responsive to the abundance (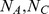), carrying capacity (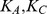), and vector competence (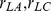) of mosquito vectors. All of these relationships were positive, with the exception that 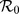 had a negative correlation with mosquito abundance due to the density dependence of birth (and correspondingly, death) rates such that higher numbers of vectors resulted in smaller values for the 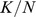 ratio, which also limited 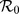. 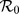 was much more sensitive than 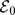 to variation in temperature that affected RVFV extrinsic incubation and vector biting rates, which explained its broader range of values within each land use class (Figure 5, lower panel). The abundance of hosts had little effect on either transmission metric. Complete results for the sensitivity analysis are presented in Mathematical Appendix Table S3 in Text S1.

## Discussion

This study extends previous models of RVFV dynamics [17]–[23], [53], [54] by developing methods for linking our model and potentially others to real landscapes. We have demonstrated these methods in a well-studied and potentially RVFV-receptive region of California with high densities of dairy cattle and mosquitoes and a history of arbovirus transmission. Where possible, we have utilized readily available continental-scale data sources, and as a result, our methods could be employed to broadly assess risk for RVFV elsewhere in North America, given adequate knowledge of local patterns in vector and host population dynamics, although realistically addressing this last point is not a trivial hurdle for any broad-scale risk assessment.

Our model modifies the parameterization of several earlier RVFV models [17]–[20], [23] that had the potential to downplay the role of vertical transmission in *Aedes*. In these models, the infected subadult stage (eggs or simply “aquatic stage”) arose by applying the probability of vertical transmission (denoted 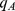 herein) to infectious adult females (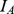). This would be appropriate, except that it implies that salivary transmission of RVFV by *Aedes* females is a necessary condition for vertical transmission to offspring. These processes occur by parallel infection pathways, although both require initial passage through the midgut barrier and a subsequent incubation period. The model we have presented continues to define infected eggs (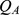) as originating from infectious females (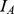), which allows for the reasonable assumption that the incubation period for horizontal and vertical transmission are equal. However, we rescaled the parameter 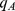 by the vector competence parameter 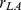 to match our understanding of the natural definition of 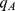, equal to the per capita probability of infection in offspring given that a female mosquito feeds on an infectious host (See Mathematical Appendix, Equation 2). Despite the conceptual aspects, previous models have assumed larger values for this vertical transmission parameter (most commonly 0.05) compared to the 0.001 we have chosen here based on the rarity of isolations from field-collected immature mosquitoes [31] and null findings in the laboratory [5], [55]–[57], which would negate some of the potential downward bias in model outcomes.

Previous dynamical models for RVFV have evaluated transmission outcomes for single parameter sets, generally combined with sensitivity analysis, but have not focused on realistic spatio-temporal variation in transmission risk for RVFV. One recent exception included two sets of parameters for “dry” vs. “wet” seasons [23], resulting in 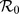 values of 0.80 and 2.28, respectively, which were within the range of our estimates (0.23–3.24). The range for 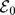 was much narrower (1.84–10.57 compared to our 0.18–95.27), which is probably due to our broader range of parameters, particularly the data-driven seasonal variation and inequality of the abundance and carrying capacity of vectors in our model. Unlike in RVFV-enzootic areas of sub-Saharan Africa [53], [58], California's rainfall occurs during the coolest months of the year and does not directly drive the population dynamics of most mosquitoes, so the deliberate distribution of surface water by humans (e.g., in irrigated agriculture and wetlands) creates and maintains aquatic mosquito habitats, and therefore transmission is expected to be greatest during the warm, “dry” season when both vector abundance and temperatures are high. 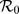 values from single-patch [17] and metapopulation models for RVFV [19] (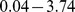 and 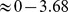, respectively) also agreed well with our estimates, although the ranges in these studies were calculated from parameter sets randomly drawn from uniform distributions rather than “real-world” scenarios.

We focused on two important transmission metrics, 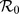 and 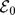, to convey the average and maximal expectations for transmission in initially disease-free, immunologically nave populations. Such a scenario is appropriate for the Western Hemisphere, which has no history of RVFV transmission, and these metrics were useful for defining the spatio-temporal range over which an introduced virus could cause outbreaks and the relative variation in risk over a typical year, which are driven primarily by seasonal patterns in vector abundance and temperature.

Our model-based estimates suggest that the risk for an RVFV outbreak – if it were to be introduced – should be a concern for California's dairy industry during all but the coldest winter months of December and January and is highest during the warm spring and summer seasons in the Central Valley. Epidemic risk increases rapidly once the abundance of mosquitoes begins to increase, typically in March–April, and 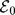 has shown that the potential for RVFV circulation begins earlier and lasts longer than would be suggested by an analysis informed by 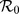 alone. The theoretical threshold of 1.0 for 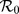 should be viewed with caution as an absolute limit for transmission, especially for vector-borne diseases [48], and its values depend on the models and methods chosen for computation [59]. However, we believe the relative patterns are more important than the exact numerical values for determining when and where the risk for RVFV outbreaks would be highest.

In parameterizing the model, we were confronted with many gaps in knowledge and data. This contrasts with our recent study [20] that illustrated a similar methodology for WNV, a system where the competent vectors are well-known and previous studies could be invoked to estimate the EIP and biting rates as functions of environmental temperature. For RVFV, the competent North American vectors are only partially known [6], and the data available for quantifying extrinsic incubation rates at different temperatures are scanty and based on colonized mosquitoes [42], [43]. Similarly, there is little data available for North American *Aedes* species on the variation in biting rates with temperature; here we have used a model based on egg maturation rates in *Culex* mosquitoes [45] and applied it to both genera of RVFV vectors. This is complicated by fact that *Aedes* may take multiple partial blood meals during a gonotrophic cycle, so a rate based on ovarian development periods may understate the frequency of vector-host contact.

In our model, we assumed that the vectors feed on the two types of hosts represented by cattle and birds at rates proportional to their availability. Most studies on *Cx. tarsalis* have found a broad host range, including mammals, with a greater number of blood meals taken on avian hosts compared to mammalian hosts [33], [36], [37], [60]–[64]. These patterns appear to be a function of host availability and possibly other factors such as body size or defensive behaviors. Studies utilizing forage ratios to relate feeding to proportionate availability have found that passerines were often underrepresented in relation to their densities [36], [63], [65], while cattle were frequently the most common – and sometimes overrepresented – mammalian host [36], [37], [60], [61], [65], [66]. Studies on *Ae. melanimon* have indicated a consistent preference for feeding on mammals over birds (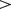
90% of total blood meals; [33], [60], [62], [66], [67]). In our study area, cows generally constituted a high proportion of the total hosts in each area (i.e., cows represented 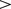
50% and 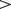
90% of all hosts in 94.5% and 59.7% of the areas modeled, respectively), which meant that the opportunistic feeding assumption resulted in consistently high rates of biting on cattle for both species. The impacts of host preferences on the potential for RVFV transmission is an interesting topic for consideration in future modeling studies.

To understand the potential role of *Aedes*, *Culex*, and possibly other genera in the transmission of RVFV or other pathogens to cattle, we need a better understanding of heterogeneities in biting pressure from the various mosquito species. Other studies have shown promise in this regard, in the use of antibodies to salivary antigens as biomarkers for vector exposure (e.g., [68]–[72]) and specifically in characterization of the sialotranscriptome for *Cx. tarsalis* [73].

There are also substantial uncertainties in the viral transmission cycle should RVFV find its way into North America. Which wildlife species would serve as competent hosts is a key unknown. White-tailed deer are very abundant in the eastern US and have been hypothesized as potential wildlife carriers in North America [13], but the ability of these animals to become infected and develop viremias high enough to infect biting vectors remains undocumented. The potential role of lagomorphs (hares and rabbits) is also unknown, although these are important in the transmission cycles of several bunyaviruses in the family *Bunyaviridae* in California [30], [34]. Our study therefore did not include wildlife hosts.

Despite the potential for RVFV outbreaks, it is unclear whether the virus could persist between years to become enzootic in the U.S. This uncertainty is due in part to the numerous gaps in the data available to inform model parameters described above, but also to the seasonality of temperatures and vector populations that ensure that conditions are never constant. Additional study is needed within a stochastic modeling framework to understand the range of potential invasion mechanisms (e.g., mosquitoes vs. human or animal hosts), as well as the potential mechanisms for RVFV persistence and whether they could permit the virus to avoid the possibility of stochastic fadeout during North America's winter. Host and vector movements will be important for short-term spread of RVFV [18], [19], but their necessity for interannual persistence will depend in part on whether the African paradigm of inter-epizootic maintenance in vertically infected mosquitoes holds true. If vertical transmision turns out to be an inadequate persistence mechanism in North American *Aedes* or *Culex*, movement could increase the likelihood of RVFV persistence by bringing infectious vectors and hosts into contact with new susceptible subpopulations [54]. Data on livestock movement are limited, but new Bayesian methods are giving hope that more can be done with incomplete data [74].

### Conclusion

We have developed novel, generalizable methods to link mathematical models for RVFV with broad-scale spatio-temporal data for realistic landscapes. These methods could be useful for prioritizing when and where to focus control strategies (e.g., vector control or cattle vaccination) during an invasion. Many gaps in both data and knowledge remain, but this is an important step toward understanding the potential seasonal transmission cycles of RVFV and other vectorborne pathogens that may invade temperate North America.

## Supporting Information

Text S1 Mathematical appendix describing the model, including definitions for state variables and parameters and results for sensitivity analysis.
(PDF)

## References

[1] Bird BH, Ksiazek TG, Nichol ST, MacLachlan NJ (2009) Rift Valley fever virus. Journal of the American Veterinary Medical Association 234: 883–893. 19335238 10.2460/javma.234.7.883

[2] Flick R, Bouloy M (2005) Rift Valley fever virus. Current Molecular Medicine 5: 827–834. 16375716 10.2174/156652405774962263

[3] Meegan J, Bailey C (1989) Rift Valley Fever. In: Monath T, editor. The Arboviruses: Epidemiology and Ecology. Vol. IV, Ch. 39. Boca Raton, FL: CRC Press. pp. 51–76.

[4] Turell M, Lee J, Richardson J, Sang R, Kioko E, et al (2007) Vector competence of Kenyan *Culex zombaensis* and *Culex quinquefasciatus* mosquitoes for Rift Valley fever virus. Journal of the American Mosquito Control Association 23: 378. 18240513 10.2987/5645.1

[5] Turell M, Linthicum K, Patrican L, Davies F, Kairo A, et al (2008) Vector competence of selected African mosquito (Diptera: Culicidae) species for Rift Valley fever virus. Journal of Medical Entomology 45: 102–108. 18283949 10.1603/0022-2585(2008)45[102:vcosam]2.0.co;2

[6] Turell M, Wilson W, Bennett K (2010) Potential for North American mosquitoes (Diptera: Culicidae) to transmit Rift Valley fever virus. Journal of Medical Entomology 47: 884–889. 20939385 10.1603/me10007

[7] Meegan J, Moussa M, el Mour A, Toppouzzada R, Wyess R (1978) Ecological and epidemiological studies of Rift Valley fever in Egypt. Journal of the Egyptian Public Health Association 53: 173–175. 752700

[8] Meegan J, Niklasson B, Bengtsson E (1979) Spread of Rift Valley fever virus from continental Africa. The Lancet 314: 1184–1185. 10.1016/s0140-6736(79)92406-1 91909

[9] US Department of Agriculture-Animal and Plant Health Inspection Service, US Centers for Disease Control and Prevention (2012). National Select Agent Registry. Internet. Available: http://www.selectagents.gov.

[10] Iranpour M, Turell M, Lindsay L (2011) Potential for Canadian mosquitoes to transmit Rift Valley fever virus. Journal of the American Mosquito Control Association 27: 363–369. 22329267 10.2987/11-6169.1

[11] Darsie Jr R, Ward R, Carpenter S, LaCasse W (2005) Identification and geographical distribution of the mosquitoes of North America, North of Mexico. University Press of Florida. 383 pp.

[12] Hartley D, Rinderknecht J, Nipp T, Clarke N, Snowder G, et al (2011) Potential effects of Rift Valley fever in the United States. Emerging Infectious Diseases 17: e1. 10.3201/eid1708.101088 PMC3381545 21801607

[13] Kakani S, LaBeaud A, King C (2010) Planning for Rift Valley fever virus: Use of GIS to estimate the human health threat of white-tailed deer (Odocoileus virginianus)-related transmission. Geospatial Health 5: 33–43. 21080319 10.4081/gh.2010.185 PMC3140430

[14] European Food Safety Authority (EFSA) (2005) Opinion of the scientific panel on animal health and welfare (AHAW) on a request from the Commission related to “The risk of a Rift Valley fever incursion and its persistence within the community”. EFSA Journal 238: 1–128.

[15] Chevalier V, Pepin M, Plee L, Lancelot R (2010) Rift valley fever-a threat for Europe? Euro Surveillance 15: 19506. 20403309

[16] Kasari T, Carr D, Lynn T, Weaver J (2008) Evaluation of pathways for release of Rift Valley fever virus into domestic ruminant livestock, ruminant wildlife, and human populations in the continental United States. Journal of the American Veterinary Medical Association 232: 514–529. 18279085 10.2460/javma.232.4.514

[17] Gaff H, Hartley D, Leahy N (2007) An epidemiological model of Rift Valley fever. Electronic Journal of Differential Equations 2007: 1–12.

[18] Niu T, Gaff HD, Papelis YE, Hartley DM (2012) An epidemiological model of Rift Valley fever with spatial dynamics. Computational and Mathematical Methods in Medicine 2012: 1–12. 10.1155/2012/138757 PMC3424773 22924058

[19] Xue L, Scott H, Cohnstaedt L, Scoglio C (2012) A network-based meta-population approach to model Rift Valley fever epidemics. Journal of Theoretical Biology 306: 129–144. 22564391 10.1016/j.jtbi.2012.04.029

[20] Gaff H, Burgess C, Jackson J, Niu T, Papelis Y, et al (2011) Mathematical model to assess the relative effectiveness of Rift Valley fever countermeasures. International Journal of Artificial Life Research 2: 1–18.

[21] Adongo D, Fister KR, Gaff H, Hartley D (2013) Optimal control applied to Rift Valley fever. Natural Resource Modeling 26: 385–402.

[22] Mpeshe S, Haario H, Tchuenche J (2011) A mathematical model of Rift Valley fever with human host. Acta Biotheoretica 59: 231–250. 21611886 10.1007/s10441-011-9132-2

[23] Chitnis N, Hyman JM, Manore CA (2013) Modelling vertical transmission in vector-borne diseases with applications to Rift Valley fever. Journal of Biological Dynamics 7: 11–40. 23098257 10.1080/17513758.2012.733427 PMC4260360

[24] Anderson RM, May RM (1992) Infectious Diseases of Humans: Dynamics and Control. Oxford University Press, USA.

[25] Hosack GR, Rossignol PA, van den Driessche P (2008) The control of vector-borne disease epidemics. Journal of Theoretical Biology 255: 16–25. 18706917 10.1016/j.jtbi.2008.07.033

[26] Reisen W, Carroll B, Takahashi R, Fang Y, Garcia S, et al (2009) Repeated West Nile virus epidemic transmission in Kern County, California, 2004–2007. Journal of Medical Entomology 46: 139–157. 19198528 10.1603/033.046.0118 PMC2729460

[27] Reeves WC (1990) Epidemiology and Control of Mosquito-borne Arboviruses in California, 1943–1987. Sacramento, CA: California Mosquito and Vector Control Association.

[28] Reisen W, Milby M, Meyer R (1992) Population dynamics of adult Culex mosquitoes (Diptera: Culicidae) along the Kern River, Kern County, California, in 1990. Journal of Medical Entomology 29: 531–543. 1625303 10.1093/jmedent/29.3.531

[29] Nemani R, Votava P, Michaelis A, White M, Melton F, et al.. (2007) Remote sensing methodologies for ecosystem management. In: Aswathanarayana U, editor. Food & Water Security. Oxford, UK: Taylor & Francis. pp. 1–19.

[30] Reisen W, Hardy J, Reeves W, Presser S, Milby M, et al (1990) Persistence of mosquito-borne viruses in Kern County, California, 1983–1988. American Journal of Tropical Medicine and Hygiene 43: 419–437. 2240370 10.4269/ajtmh.1990.43.419

[31] Linthicum K, Davies F, Kairo A, Bailey C, et al (1985) Rift Valley fever virus (family *Bunyaviridae*, genus *Phlebovirus*). Isolations from Diptera collected during an inter-epizootic period in Kenya. Journal of Hygiene (London) 95: 197–209. 10.1017/s0022172400062434 PMC2129511 2862206

[32] Turell M, Reeves W, Hardy J (1982) Transovarial and trans-stadial transmission of California encephalitis virus in *Aedes dorsalis* and *Aedes melanimon* . American Journal of Tropical Medicine and Hygiene 31: 1021–1029. 6889818 10.4269/ajtmh.1982.31.1021

[33] Reisen WK, Reeves WC (1990) Bionomics and ecology of *Culex tarsalis* and other potential mosquito vector species. In: Epidemiology and Control of Mosquito-borne Arboviruses in California, 1943–1987. Sacramento, CA: California Mosquito and Vector Control Assoc. pp. 254–329.

[34] Hardy JL, Reeves WC, Reeves WC (1990) Experimental studies on infection in vectors. In: Epidemiology and Control of Mosquito-borne Arboviruses in California, 1943–1987. Sacramento, California: California Mosquito and Vector Control Association. pp. 145–250.

[35] Reisen W, Fang Y, Martinez V (2005) Avian host and mosquito (Diptera: Culicidae) vector competence determine the efficiency of West Nile and St. Louis encephalitis virus transmission. Journal of Medical Entomology 42: 367–375. 15962789 10.1093/jmedent/42.3.367

[36] Thiemann T, Wheeler S, Barker C, Reisen W (2011) Mosquito host selection varies seasonally with host availability and mosquito density. PLoS Neglected Tropical Diseases 5: e1452. 22206038 10.1371/journal.pntd.0001452 PMC3243726

[37] Thiemann T, Lemenager D, Kluh S, Carroll B, Lothrop H, et al (2012) Spatial variation in host feeding patterns of *Culex tarsalis* and the *Culex pipiens* complex (Diptera: Culicidae) in California. Journal of Medical Entomology 49: 903–916. 22897051 10.1603/me11272 PMC3542768

[38] Newhouse V, Chamberlain R, Johnston J, Sudia W (1966) Use of dry ice to increase mosquito catches of the CDC miniature light trap. Mosquito News 26: 30–35.

[39] Meegan JM (1979) The Rift Valley fever epizootic in Egypt 1977–1978 1. description of the epizootic and virological studies. Transactions of the Royal Society of Tropical Medicine and Hygiene 73: 618–623. 10.1016/0035-9203(79)90004-x 538803

[40] Simons T, Alldredge M, Pollock K, Wettroth J, Dufty A Jr (2007) Experimental analysis of the auditory detection process on avian point counts. The Auk 124: 986–999.

[41] Hartley DM, Barker CM, Le Menach A, Niu T, Gaff HD, et al (2012) Effects of temperature on emergence and seasonality of West Nile virus in California. American Journal of Tropical Medicine and Hygiene 86: 884–894. 22556092 10.4269/ajtmh.2012.11-0342 PMC3335698

[42] Turell M, Rossi C, Bailey C (1985) Effect of extrinsic incubation temperature on the ability of Aedes taeniorhynchus and Culex pipiens to transmit Rift Valley fever virus. American Journal of Tropical Medicine and Hygiene 34: 1211–1218. 3834803 10.4269/ajtmh.1985.34.1211

[43] Turell M (1989) Effect of environmental temperature on the vector competence of Aedes fowleri for Rift Valley fever virus. Research in Virology 140: 147–154. 2756242 10.1016/s0923-2516(89)80092-5

[44] Brubaker JF, Turell MJ (1998) Effect of environmental temperature on the susceptibility of Culex pipiens (Diptera: Culicidae) to Rift Valley fever virus. Journal of Medical Entomology 35: 918–921. 9835680 10.1093/jmedent/35.6.918

[45] Reisen W, Milby M, Presser S, Hardy J (1992) Ecology of mosquitoes and St. Louis encephalitis virus in the Los Angeles basin of California, 1987–1990. Journal of Medical Entomology 29: 582–598. 1495066 10.1093/jmedent/29.4.582

[46] van den Driessche P, Watmough J (2002) Reproduction numbers and sub-threshold endemic equilibria for compartmental models of disease transmission. Mathematical Biosciences 180: 29–48. 12387915 10.1016/s0025-5564(02)00108-6

[47] Heffernan JM, Smith RJ, Wahl LM (2005) Perspectives on the basic reproductive ratio. Journal of the Royal Society Interface 2: 281–93. 10.1098/rsif.2005.0042 PMC1578275 16849186

[48] Massad E, Coutinho FAB, Burattini MN, Amaku M Estimation of R_0_ from the initial phase of an outbreak of a vector-borne infection. Tropical Medicine & International Health 15: 120–126. 10.1111/j.1365-3156.2009.02413.x 19891761

[49] Dushoff J, Huang WZ, Castillo-Chavez C Backwards bifurcations and catastrophe in simple models of fatal diseases. Journal of Mathematical Biology 36: 227–248. 9528119 10.1007/s002850050099

[50] Lipsitch M, Nowak MA, Ebert D, May RM (1995) The population dynamics of vertically and horizontally transmitted parasites. Proceedings of the Royal Society of London Series B: Biological Sciences 260: 321–327. 7630898 10.1098/rspb.1995.0099

[51] Matala A (2008) Sample size requirement for Monte Carlo simulations using Latin hypercube sampling. Mat-24108, Helsinki Univ of Techology.

[52] R Core Team (2012). R: A language and environment for statistical computing. URL http://www.R-project.org/.

[53] Bicout D, Sabatier P (2004) Mapping Rift Valley fever vectors and prevalence using rainfall variations. Vector-borne and Zoonotic Diseases 4: 33–42. 15018771 10.1089/153036604773082979

[54] Favier C, Chalvet-Monfray K, Sabatier P, Lancelot R, Fontenille D, et al (2006) Rift Valley fever in West Africa: the role of space in endemicity. Tropical Medicine & International Health 11: 1878–1888. 17176353 10.1111/j.1365-3156.2006.01746.x

[55] McIntosh B, Jupp P, Dos Santos I, Barnard B (1980) Vector studies on Rift Valley fever virus in South Africa. South African Medical Journal 58: 127–132. 6105722

[56] McIntosh B, Jupp P (1981) Epidemiological aspects of Rift Valley fever in South Africa with reference to vectors. Contributions to Epidemiology and Biostatistics 3: 92–99.

[57] Turell MJ, Faran ME, Cornet M, Bailey CL (1988) Vector competence of Senegalese *Aedes fowleri* (Diptera: Culicidae) for Rift Valley fever virus. Journal of Medical Entomology 25: 262–266. 3404545 10.1093/jmedent/25.4.262

[58] Linthicum KJ, Anyamba A, Tucker CJ, Kelley PW, Myers MF, et al (1999) Climate and satellite indicators to forecast Rift Valley fever epidemics in Kenya. Science 285: 397–400. 10411500 10.1126/science.285.5426.397

[59] Li J, Blakeley D, Smith RJ (2011) The failure of R0. Computational and Mathematical Methods in Medicine 2011: 1–17. 10.1155/2011/527610 PMC3157160 21860658

[60] Reeves WC, Hammon WM (1962) Epidemiology of the Arthropod-borne Viral Encephalitides in Kern County, California, 1943–1952. Vol. 4. University of California Press. 14491029

[61] Reeves W, Tempelis C, Bellamy R, Lofy M (1963) Observations on the feeding habits of *Culex tarsalis* in Kern County, California, using precipitating antisera produced in birds. American Journal of Tropical Medicine and Hygiene 12: 929–935. 14075022 10.4269/ajtmh.1963.12.929

[62] Tempelis G, Washino R (1967) Host-feeding patterns of *Culex tarsalis* in the Sacramento Valley, California, with notes on other species. Journal of Medical Entomology 4: 315–318. 4383195 10.1093/jmedent/4.3.315

[63] Kent R, Juliusson L, Weissmann M, Evans S, Komar N (2009) Seasonal blood-feeding behavior of *Culex tarsalis* (Diptera: Culicidae) in Weld County, Colorado, 2007. Journal of Medical Entomology 46: 380–390. 19351092 10.1603/033.046.0226

[64] Molaei G, Cummings RF, Su T, Armstrong PM, Williams GA, et al (2010) Vector-host interactions governing epidemiology of West Nile virus in Southern California. American Journal of Tropical Medicine and Hygiene 83: 1269–1282. 21118934 10.4269/ajtmh.2010.10-0392 PMC2990044

[65] Hayes RO, Tempelis CH, Hess AD, Reeves WC (1973) Mosquito host preference studies in Hale County, Texas. American Journal of Tropical Medicine and Hygiene 22: 270–277. 4143926 10.4269/ajtmh.1973.22.270

[66] Tempelis C, Francy D, Hayes R, Lofy MF (1967) Variations in feeding patterns of seven culicine mosquitoes on vertebrate hosts in Weld and Larimer Counties, Colorado. American Journal of Tropical Medicine and Hygiene 16: 111–119. 4381479 10.4269/ajtmh.1967.16.111

[67] Tempelis C (1975) Host-feeding patterns of mosquitoes, with a review of advances in analysis of blood meals by serology. Journal of Medical Entomology 11: 635–653. 235647 10.1093/jmedent/11.6.635

[68] Trevejo RT, Reeves WC (2005) Antibody response to Culex tarsalis salivary gland antigens among sentinel chickens in California. American Journal of Tropical Medicine and Hygiene 72: 481–487. 15827292

[69] Trevejo RT, Reisen WK, Yoshimura G, Reeves WC (2005) Detection of chicken antibodies to mosquito salivary gland antigens by enzyme immunoassay. Journal of the American Mosquito Control Association 21: 39–48. 15825760 10.2987/8756-971X(2005)21[39:DOCATM]2.0.CO;2

[70] Remoué F, Cisse B, Ba F, Sokhna C, Herve J, et al (2006) Evaluation of the antibody response to *Anopheles* salivary antigens as a potential marker of risk of malaria. Transactions of the Royal Society of Tropical Medicine and Hygiene 100: 363–370. 16310235 10.1016/j.trstmh.2005.06.032

[71] Orlandi-Pradines E, Almeras L, Denis de Senneville L, Barbe S, Remoué F, et al (2007) Antibody response against saliva antigens of *Anopheles gambiae* and *Aedes aegypti* in travellers in tropical Africa. Microbes and Infection 9: 1454–1462. 17913537 10.1016/j.micinf.2007.07.012

[72] Cornelie S, Remoue F, Doucoure S, NDiaye T, Sauvage FX, et al (2007) An insight into immunogenic salivary proteins of *Anopheles gambiae* in African children. Malaria Journal 6: 75. 17550586 10.1186/1475-2875-6-75 PMC1891310

[73] Calvo E, Sanchez-Vargas I, Favreau AJ, Barbian KD, Pham VM, et al (2010) An insight into the sialotranscriptome of the West Nile mosquito vector, *Culex tarsalis* . BMC Genomics 11: 51. 20089177 10.1186/1471-2164-11-51 PMC2823692

[74] Lindström T, Grear DA, Buhnerkempe M, Webb CT, Miller RS, et al (2013) A Bayesian approach for modeling cattle movements in the United States: Scaling up a partially observed network. PLoS ONE 8: e53432. 23308223 10.1371/journal.pone.0053432 PMC3537632

