# Supplementary material for: Data-Driven Modeling to Assess Receptivity for Rift Valley Fever Virus
Source: PLoS Negl Trop Dis. 2013 Nov 14;7(11):e2515. doi: 10.1371/journal.pntd.0002515 (PMC3828160; doi:10.1371/journal.pntd.0002515)
Supplement: Text S1 — Mathematical appendix describing the model, including definitions for state variables and parameters and results for sensitivity analysis. (PDF) [file pntd.0002515.s001.pdf]

1 Mathematical Appendix

1.1 Model Description

The population of *Aedes* vectors consists of uninfected (P_A) and infected (Q_A) eggs, and susceptible (S_A), incubating (E_A; infected, but not yet infectious) and infectious (I_A) adult individuals. The size of the adult *Aedes* mosquito population is $N_A = S_A + E_A + I_A$. The population of adult *Culex* vectors consists of susceptible (S_C), incubating (E_C) and infectious (I_C) adult individuals. The size of adult *Culex* mosquito population is $N_C = S_C + E_C + I_C$. The aquatic population of *Culex* only has uninfected (P_C) eggs because we assume there is no vertical transmission of RVFV in *Culex*. The livestock populations consist of susceptible (S_L), incubating (E_L), infectious (I_L), and immune (R_L) individuals. The total livestock population size is $N_L = S_L + E_L + I_L + R_L$. The other host population serves as a sink, potentially diverting mosquito bites from competent hosts, and the total population size for these sink hosts is N_D. The resulting system of the ordinary differential equations is:

Vector 1 (A): *Aedes* mosquitoes with vertical transmission:

$$\frac{dP_A}{dt} = \frac{b_A K_A}{N_A}(N_A - q_A I_A) - \frac{b_A K_A}{N_A} P_A \tag{1}$$

$$\frac{dQ_A}{dt} = \frac{b_A K_A}{N_A}(\frac{q_A}{r_{LA}} I_A - Q_A) \tag{2}$$

$$\frac{dS_A}{dt} = \frac{b_A K_A}{N_A} P_A - \beta_{LA} S_A \frac{I_L}{N_L} - \frac{d_A N_A}{K_A} S_A \tag{3}$$

$$\frac{dE_A}{dt} = \beta_{LA} S_A \frac{I_L}{N_L} - \frac{d_A N_A}{K_A} E_A - \varepsilon_A E_A \tag{4}$$

$$\frac{dI_A}{dt} = \frac{b_A K_A}{N_A} Q_A + \varepsilon_A E_A - \frac{d_A N_A}{K_A} I_A \tag{5}$$

$$\frac{dN_A}{dt} = \frac{b_A K_A}{N_A}(P_A + Q_A) - \frac{d_A N_A}{K_A} N_A \tag{6}$$

Vector 2 (C): *Culex* mosquitoes:

$$\frac{dP_C}{dt} = \frac{b_C K_C}{N_C} N_C - \frac{b_C K_C}{N_C} P_C \quad (7)$$

$$\frac{dS_C}{dt} = \frac{b_C K_C}{N_C} P_C - \beta_{LC} S_C \frac{I_L}{N_L} - \frac{d_C N_C}{K_C} S_C \quad (8)$$

$$\frac{dE_C}{dt} = \beta_{LC} S_C \frac{I_C}{N_C} - \frac{d_C N_C}{K_C} E_C - \varepsilon_C E_C \quad (9)$$

$$\frac{dI_C}{dt} = \varepsilon_C E_C - \frac{d_C N_C}{K_C} I_C \quad (10)$$

$$\frac{dN_C}{dt} = \frac{b_C K_C}{N_C} P_C - \frac{d_C N_C}{K_C} N_C \quad (11)$$

Livestock host (L):

$$\frac{dS_L}{dt} = b_L N_L - \beta_{AL} S_L \frac{I_A}{N_A} - \beta_{CL} S_L \frac{I_C}{N_C} - \frac{d_L N_L}{K_L} S_L \quad (12)$$

$$\frac{dE_L}{dt} = \beta_{AL} S_L \frac{I_A}{N_A} + \beta_{CL} S_L \frac{I_C}{N_C} - \frac{d_L N_L}{K_L} E_L - \varepsilon_L E_L \quad (13)$$

$$\frac{dI_L}{dt} = \varepsilon_L E_L - (\mu_L + \gamma_L) I_L - \frac{d_L N_L}{K_L} I_L \quad (14)$$

$$\frac{dR_L}{dt} = \gamma_L I_L - \frac{d_L N_L}{K_L} R_L \quad (15)$$

$$\frac{dN_L}{dt} = b_L N_L - \frac{d_L {N_L}^2}{K_L} - \mu_L I_L \quad (16)$$

Dead-end host (D):

$$\frac{dN_D}{dt} = b_D N_D - \frac{d_D {N_D}^2}{K_D} \quad (17)$$

Table S1. Definitions for model parameters.

Symbol	Description	Definition	Reference
b_A	Birth rate of *Aedes* vectors	$= d_A$	
b_C	Birth rate of *Culex* vectors	$= d_C$	
b_L	Birth rate of livestock	$= d_L$	
b_D	Birth rate of dead-end hosts	$= d_D$	
K_A	Carrying capacity for *Aedes* vectors	$(\frac{Aedes\ females}{CO_2\ trap-night})_{time+1} \times GP_A \times (N_L + N_D)$	

K_C	Carrying capacity for *Culex* vectors	$(\frac{Culex\ females}{CO_2\ trap-night})_{time+1} \times GP_C \times (N_L + N_D)$	
K_L	Carrying capacity for livestock	$= N_L$ (from data)	(see text & Table S2)
K_D	Carrying capacity for dead-end hosts	$= N_D$ (from data)	(see text & Table S2)
$1/d_A$	Life span of *Aedes* vectors	10 days	[1]
$1/d_C$	Life span of *Culex* vectors	10 days	[2, 3]
$1/d_L$	Life span of livestock	5 years	
$1/d_D$	Life span of dead-end hosts	1 year	[4, 5]
q_A	Probability of vertical transmission	0.001	[6, 7]
β_{LA}	Transmission rate from livestock to *Aedes*	$\frac{r_{LA}}{GP_A} \times f_L$	
β_{LC}	Transmission rate from livestock to *Culex*	$\frac{r_{LC}}{GP_C} \times f_L$	
β_{AL}	Transmission rate from *Aedes* to livestock	$\beta_{LA}\frac{N_A}{N_L}$	
β_{CL}	Transmission rate from *Culex* to livestock	$\beta_{LC}\frac{N_C}{N_L}$	
r_{LA}	Probability of transmission from livestock to *Aedes* per bite	0.005	[8]
r_{LC}	Probability of transmission from livestock to *Culex* per bite	0.09	[8]
GP_A	Gonotrophic period for *Aedes* in days	$2 + (-0.066 + 0.018T)^{-1}$	[9]
GP_C	Gonotrophic period for *Culex* in days	$2 + (-0.066 + 0.018T)^{-1}$	[9]

f_L	Probability of mosquito feeding on livestock	$\frac{N_L}{N_L+N_D}$	
$1/\varepsilon_A$	Extrinsic incubation period of RVFV in *Aedes*	$(-0.1038+0.0071T)^{-1}$	[10, 11]; but see methods
$1/\varepsilon_C$	Extrinsic incubation period of RVFV in *Culex*	$(-0.1038+0.0071T)^{-1}$	[10, 11]; but see methods
$1/\varepsilon_L$	Intrinsic incubation period of RVFV in livestock	1 day	[12]
T	Temperature (°C)	10-year mean daily temperatures, 2002–2011	TOPS [13]
$1/\gamma_L$	Infectious period of livestock	4 days	[14]
$1/\mu_L$	Excess disease-related mortality of livestock	$-log(1-0.25)\times\gamma_L$	[14]

Table S2. Data-based definitions for state variables for each grid cell.[a]

Symbol	Description	Definition	Source
N_A	Number of *Aedes* vectors	$(\frac{Aedes\ females}{CO_2\ trap-night})_{time}\times GP_A\times(N_L+N_D)$	CO_2 trap records from mosquito control agencies linked to land use classes defined in the National Land Cover Data Set 2006
N_C	Number of *Culex* vectors	$(\frac{Culex\ females}{CO_2\ trap-night})_{time}\times GP_C\times(N_L+N_D)$	CO_2 trap records from mosquito control agencies linked to land use classes defined in the National Land Cover Data Set 2006
N_L	Number of livestock	Direct tabulation from spatial data	California Water Resources Control Board
N_D	Number of dead-end hosts	$\left(\frac{Birds}{Survey\ route}\right)\times\left(\frac{25}{50\pi 0.2}\right)$	USGS Breeding Bird Survey

[a] See the methods section for further explanation.

1.2 Computation of $\mathcal{R}_0$

$\mathcal{R}_0$ is computed according to the next-generation matrices method described by van den Driessche and Watmough [15], and the details are summarized here for completeness. For vertical transmission, the next-generation matrices (F_V and V_V) are as below:

$$F_V = \begin{bmatrix} 0 & \frac{b_A K_A}{N_A}\frac{q_A}{r_{LA}} \\ 0 & 0 \end{bmatrix} \tag{18}$$

$$V_V = \begin{bmatrix} \frac{b_A K_A}{N_A} & 0 \\ -\frac{b_A K_A}{N_A} & \frac{d_A N_A}{K_A} \end{bmatrix} \tag{19}$$

Let $x_j = \frac{X_j}{N_j}, j = A, C, L$ and $X = S, E, I, K$. Then the next-generation matrices of the horizontal transmission (F_H and V_H) are:

$$F_H = \begin{bmatrix} 0 & 0 & 0 & \beta_{LA}s_A & 0 & 0 \\ 0 & 0 & 0 & 0 & 0 & 0 \\ 0 & \beta_{AL}s_L & 0 & 0 & 0 & \beta_{CL}s_L \\ 0 & 0 & 0 & 0 & 0 & 0 \\ 0 & 0 & 0 & \beta_{LC}s_C & 0 & 0 \\ 0 & 0 & 0 & 0 & 0 & 0 \end{bmatrix} \tag{20}$$

$$V_H = \begin{bmatrix} d_A/k_A + \varepsilon_A & 0 & 0 & \beta_{LA}s_A & 0 & 0 \\ -\varepsilon_A & d_A/k_A & 0 & 0 & 0 & 0 \\ 0 & 0 & d_L/k_L + \varepsilon_L & 0 & 0 & 0 \\ 0 & 0 & -\varepsilon_L & d_L/k_L + \mu_L + \gamma_L & 0 & 0 \\ 0 & 0 & 0 & 0 & d_C/k_C + \varepsilon_C & 0 \\ 0 & 0 & 0 & 0 & -\varepsilon_C & d_C/k_C \end{bmatrix} \tag{21}$$

$\mathcal{R}_0$ can be calculated as:

$$\mathcal{R}_0 = \rho\left(F_V {V_V}^{-1}\right) + \rho\left(F_H {V_H}^{-1}\right), \tag{22}$$

where ρ(matrix) represents the maximum eigenvalue of the matrix.

At disease-free equilibrium (DFE), $s_A = 1, s_C = 1, s_L = 1$. And $k_A = \frac{K_A}{N_A}, k_C = \frac{K_C}{N_C}, k_L = \frac{K_L}{N_L}$ were computed from vector and livestock data as in Tables S1 and S2.

1.3 Computation of $\mathcal{E}_0$

$\mathcal{E}_0$ was computed according to the method described by Hosack et al. [16]. $\mathcal{E}_0$ was generated from the Hermitian parts of the next-generation matrices used for the $\mathcal{R}_0$ computation as follows:

$$\begin{aligned} \mathcal{E}_0 &= \rho\left(H(F_V)H(V_V)^{-1}\right) + \rho\left(H(F_H)H(V_H)^{-1}\right) \\ &= \rho\left(\frac{F_V + F_V^t}{2} \cdot \left(\frac{V_V + V_V^t}{2}\right)^{-1}\right) + \rho\left(\frac{F_H + F_H^t}{2} \cdot \left(\frac{V_H + V_H^t}{2}\right)^{-1}\right), \end{aligned} \tag{23}$$

where ρ(matrix) represents the maximum eigenvalue of the matrix.

At the disease-free equilibrium (DFE), $s_A = 1, s_C = 1, s_L = 1$. And $k_A = \frac{K_A}{N_A}, k_C = \frac{K_C}{N_C}, k_L = \frac{K_L}{N_L}$ were computed from vector and livestock data as in Tables S1 and S2.

1.4 Sensitivity analysis for $\mathcal{R}_0$ and $\mathcal{E}_0$

The following Table S3 shows the detailed results of the sensitivity analysis.

Table S3. Ranges of parameters and their partial rank correlation coefficient (PRCC) and p-values in relation to $\mathcal{R}_0$ and $\mathcal{E}_0$.

Symbol	Range	To $\mathcal{R}_0$		To $\mathcal{E}_0$	
		PRCC	p-value	PRCC	p-value
$d_A(=b_A)$	$\left[\frac{1}{21},\frac{1}{7}\right]$	−4.7826	$< 0.0001^{***}$	−3.4486	0.0007^{**}
$d_C(=b_C)$	$\left[\frac{1}{21},\frac{1}{7}\right]$	−1.2888	0.1990	−3.7628	0.0002^{**}
$d_L(=b_L)$	$\left[\frac{1}{10\times365},\frac{1}{1\times365}\right]$	0.3857	0.7002	−0.1829	0.8550
$d_D(=b_D)$	$\left[\frac{1}{3.0\times365},\frac{1}{0.5\times365}\right]$	1.0375	0.3007	1.3555	0.1768
N_A	$(0,1000]$	−5.1767	$< 0.0001^{***}$	5.8943	$< 0.0001^{***}$
K_A	$(0,1000]$	8.4685	$< 0.0001^{***}$	6.9289	$< 0.0001^{***}$
N_C	$(0,1000]$	−5.5447	$< 0.0001^{***}$	7.3618	$< 0.0001^{***}$
K_C	$(0,1000]$	5.1922	$< 0.0001^{***}$	6.8351	$< 0.0001^{***}$
$N_L(=K_L)$	$[144,25130]$	−0.6223	0.5345	−0.6508	0.5160
$N_D(=K_D)$	$[21,262]$	−1.0991	0.2731	−0.7998	0.4248
q_A	$[0.001,0.008]$	0.2612	0.7942	1.3173	0.1893
r_{LA}	$[0.001,0.70]$	8.7827	$< 0.0001^{***}$	11.9349	$< 0.0001^{***}$
r_{LC}	$[0.001,0.70]$	6.8898	$< 0.0001^{***}$	11.9748	$< 0.0001^{***}$
ε_L	$\left[\frac{1}{6},1\right]$	−0.8097	0.4191	−7.5587	$< 0.0001^{***}$
γ_L	$\left[\frac{1}{5},1\right]$	−4.0321	0.0001^{**}	−3.6577	0.0003^{**}
μ_L	$[0.025,0.1]$	−0.9156	0.3610	−0.2306	0.8179
T	$[6.97°\text{C},31.79°\text{C}]$	16.2418	$< 0.0001^{***}$	2.6719	0.0082^{*}

*: significant at 0.01 level
**: significant at 0.001 level
***: significant at 0.0001 level

References

1. Jensen T, Washino R (1991) An assessment of the biological capacity of a Sacramento Valley population of *Aedes melanimon* to vector arboviruses. American Journal of Tropical Medicine and Hygiene 44: 355–363.

2. Reisen WK, Reeves WC (1990) Bionomics and ecology of *Culex tarsalis* and other potential mosquito vector species. In: Epidemiology and Control of Mosquito-borne Arboviruses in California, 1943–1987, Sacramento, CA: California Mosquito and Vector Control Assoc., volume 1. pp. 254–329.

3. Reisen W, Milby M, Reeves W, Meyer R, Bock M (1983) Population ecology of *Culex tarsalis* (Diptera: Culicidae) in a foothill environment of Kern County, California: temporal changes in female relative abundance, reproductive status, and survivorship. Annals of the Entomological Society of America 76: 800–808.

4. Fankhauser D (1971) Annual adult survival rates of blackbirds and starlings. Bird-Banding 42: 36–42.

5. Searcy W, Yasukawa K (1981) Sexual size dimorphism and survival of male and female blackbirds (Icteridae). The Auk : 457–465.

6. Linthicum K, Davies F, Kairo A, Bailey C, et al. (1985) Rift Valley fever virus (family Bunyaviridae, genus *Phlebovirus*). Isolations from Diptera collected during an inter-epizootic period in Kenya. Journal of Hygiene (London) 95: 197–209.

7. Turell M, Linthicum K, Patrican L, Davies F, Kairo A, et al. (2008) Vector competence of selected African mosquito (Diptera: Culicidae) species for Rift Valley fever virus. Journal of Medical Entomology 45: 102–108.

8. Turell M, Wilson W, Bennett K (2010) Potential for North American mosquitoes (Diptera: Culicidae) to transmit Rift Valley fever virus. Journal of Medical Entomology 47: 884–889.

9. Reisen W, Milby M, Presser S, Hardy J (1992) Ecology of mosquitoes and St. Louis encephalitis virus in the Los Angeles basin of California, 1987–1990. Journal of Medical Entomology 29: 582–598.

10. Turell M, Rossi C, Bailey C (1985) Effect of extrinsic incubation temperature on the ability of *Aedes taeniorhynchus* and *Culex pipiens* to transmit Rift Valley fever virus. American Journal of Tropical Medicine and Hygiene 34: 1211–1218.

11. Turell M (1989) Effect of environmental temperature on the vector competence of *Aedes fowleri* for Rift Valley fever virus. Research in Virology 140: 147–154.

12. Nfon CK, Marszal P, Zhang S, Weingartl HM (2012) Innate immune response to Rift Valley fever virus in goats. PLoS Negl Trop Dis 6: e1623.

13. Nemani R, Votava P, Michaelis A, White M, Melton F, et al. (2007) Remote sensing methodologies for ecosystem management. In: Aswathanarayana U, editor, Food & Water Security, Oxford, UK: Taylor & Francis. pp. 1-19.

14. Erasmus B, Coetzer J (1981) The symptomatology and pathology of Rift Valley fever in domestic animals. Contributions to Epidemiology and Biostatistics 3: 77–82.

15. van den Driessche P, Watmough J (2002) Reproduction numbers and sub-threshold endemic equilibria for compartmental models of disease transmission. Mathematical Biosciences 180: 29 - 48.

16. Hosack GR, Rossignol PA, van den Driessche P (2008) The control of vector-borne disease epidemics. Journal of Theoretical Biology 255: 16–25.
